# Anterior mitral valve leaflet length and response to mavacamten in obstructive hypertrophic cardiomyopathy

**DOI:** 10.1093/ehjimp/qyaf081

**Published:** 2025-06-12

**Authors:** Danish Saleh, Ellis Y Kim, Kifah Hussain, Vinesh Appadurai, Kayla Mueller, Abigail Garza, Baljash Cheema, Dominic E Fullenkamp, Vera H Rigolin, Akhil Narang, Paul C Cremer, Lubna Choudhury

**Affiliations:** Division of Cardiology, Department of Medicine, Northwestern University, Feinberg School of Medicine, Chicago, IL 60611, USA; Division of Cardiology, Department of Medicine, Northwestern University, Feinberg School of Medicine, Chicago, IL 60611, USA; Division of Cardiology, Department of Medicine, Northwestern University, Feinberg School of Medicine, Chicago, IL 60611, USA; Division of Cardiology, Department of Medicine, Northwestern University, Feinberg School of Medicine, Chicago, IL 60611, USA; School of Medicine, The University of Queensland, Queensland, Australia; The Hypertrophic Cardiomyopathy Program at the Bluhm Cardiovascular Institute, Chicago, IL 60611, USA; The Hypertrophic Cardiomyopathy Program at the Bluhm Cardiovascular Institute, Chicago, IL 60611, USA; Division of Cardiology, Department of Medicine, Northwestern University, Feinberg School of Medicine, Chicago, IL 60611, USA; The Hypertrophic Cardiomyopathy Program at the Bluhm Cardiovascular Institute, Chicago, IL 60611, USA; Division of Cardiology, Department of Medicine, Northwestern University, Feinberg School of Medicine, Chicago, IL 60611, USA; The Hypertrophic Cardiomyopathy Program at the Bluhm Cardiovascular Institute, Chicago, IL 60611, USA; Division of Cardiology, Department of Medicine, Northwestern University, Feinberg School of Medicine, Chicago, IL 60611, USA; The Hypertrophic Cardiomyopathy Program at the Bluhm Cardiovascular Institute, Chicago, IL 60611, USA; Division of Cardiology, Department of Medicine, Northwestern University, Feinberg School of Medicine, Chicago, IL 60611, USA; The Hypertrophic Cardiomyopathy Program at the Bluhm Cardiovascular Institute, Chicago, IL 60611, USA; Division of Cardiology, Department of Medicine, Northwestern University, Feinberg School of Medicine, Chicago, IL 60611, USA; The Hypertrophic Cardiomyopathy Program at the Bluhm Cardiovascular Institute, Chicago, IL 60611, USA; Division of Cardiology, Department of Medicine, Northwestern University, Feinberg School of Medicine, Chicago, IL 60611, USA; The Hypertrophic Cardiomyopathy Program at the Bluhm Cardiovascular Institute, Chicago, IL 60611, USA

**Keywords:** hypertrophic cardiomyopathy, obstructive hypertrophic cardiomyopathy, myosin inhibitors, mavacamten, anterior mitral valve leaflet, left-ventricular outflow tract

## Abstract

**Objectives:**

This study examines whether anterior mitral valve leaflet (AMVL) length is associated with response to mavacamten in patients with obstructive hypertrophic cardiomyopathy (HCM).

**Aims:**

Obstruction of the left-ventricular outflow tract (LVOT) in HCM has been associated with asymmetric septal hypertrophy and abnormalities of the mitral valve and sub-valvular apparatus. Mavacamten is a myosin-inhibitor shown to decrease LVOT gradient and improve functional status in patients with obstructive HCM.

**Methods and results:**

Measurements of cardiac structural elements were obtained from magnetic resonance imaging and echocardiography data among patients with obstructive HCM treated with mavacamten. Endpoints were effective mavacamten dose, defined as the dose required to achieve a Valsalva LVOT gradient <30 mmHg, and rapid response to mavacamten therapy, defined as achieved Valsalva LVOT gradient <20 mmHg within 8 weeks of initiation. Among 33 patients, patients with an effective dose of 5 mg (*n* = 13) had a shorter AMVL length [20.00 (18.50, 20.80) mm] compared with patients with a dose of 10 mg (*n* = 12) [23.30 (22.45, 26.10) mm] and 15 mg (*n* = 8) [25.45 (24.20, 26.85) mm] (*P* < 0.001). After adjusting for age and sex, the 5 mg dose was associated with a shorter AMVL length (*P* = 0.003). AMVL length was shorter in rapid responders [20.9 (19.9, 22.5) mm] compared with patients without a rapid response [24.9 (23.3, 26.5) mm] (*P* = 0.006).

**Conclusion:**

Shorter AMVL length is associated with a lower effective dose and a rapid response to mavacamten. If confirmed in larger studies, AMVL length may inform optimal dosing of myosin inhibitors in obstructive HCM.

## Introduction

Hypertrophic cardiomyopathy (HCM) is the most common genetic cardiomyopathy and impacts more than 20-million people worldwide.^1,2^ Dynamic left ventricular outflow tract (LVOT) obstruction is an important cause of functional limitation and heart failure in patients with HCM.^3^ In obstructive HCM, this haemodynamic perturbation is related to asymmetric interventricular septal thickness as well as abnormalities of the mitral valve and subvalvular apparatus, including elongation of the anterior mitral valve leaflet (AMVL), anteroapically displaced and thickened papillary muscles, and bifid or multi-headed papillary muscle morphology.^4–7^ These features may contribute to systolic anterior motion (SAM) of the AMVL and LVOT obstruction.^8^ Outflow tract gradients of more than 30 mmHg at rest and 50 mmHg with provocation are regarded as clinically significant and warrant consideration of medical, interventional, and surgical therapies in the symptomatic patient.^2^

Myosin-inhibitors have emerged as a novel therapy to attenuate left ventricular contractility in patients with obstructive HCM. These small molecules inhibit myosin adenosine triphosphatase activity and thereby reduce actin-myosin cross-bridging within the sarcomere.^9–11^ Mavacamten is the first commercially available myosin-inhibitor and has been shown to decrease LVOT obstruction, improve functional status, and decrease the need for septal reduction therapy in patients with obstructive HCM.^12–14^ However, obstructive HCM is a structurally heterogenous pathology. Patients with mild septal thickening can have marked LVOT gradients attributable to anterior displacement of papillary muscle positioning or aberrations within the mitral valve and subvalvular apparatus. Conversely, severe septal thickening may produce outflow tract obstruction in the absence of irregularities associated with the mitral valve.^2,5,6,15^ Accordingly, response to myosin inhibition in obstructive HCM could be related to these underlying structural abnormalities. Therefore, the aim of this study is to assess whether AMVL length is associated with the effective dose of mavacamten and rapid response to mavacamten therapy.

## Methods

### Study population and endpoints

In this single-centre retrospective cohort study, patients currently or previously treated with mavacamten were identified from the Risk Evaluation and Mitigation Strategy (REMS) enrolment at Northwestern University (*N* = 46). Mavacamten dosing was co-ordinated as previously described in the prescribing information literature issued by Bristol Myers Squibb (BMS).^16^ Two study endpoints were examined: (i) effective mavacamten dose, defined as the dose required to achieve a Valsalva LVOT gradient <30 mmHg after at least 12-weeks of therapy (‘maintenance phase’ of the REMS protocol); (ii) rapid response to mavacamten therapy, defined as achieving a Valsalva LVOT gradient <20 mmHg by week 8 (‘initiation phase’ of the REMS protocol). For analyses related to effective mavacamten dose, patient data were stratified by dose of mavacamten therapy required to achieve resolution of outflow tract obstruction (high dose = 15 mg daily, intermediate dose = 10 mg daily, low dose ≤5 mg daily). Three patients in the high dose arm did not achieve the target LVOT gradient. Prior studies have demonstrated that therapeutic dosing levels are unlikely to change over long-term follow up.^13,17^ The average follow-up time was 65 weeks, and the median follow-up time was 70 weeks. Left-ventricular dysfunction requiring discontinuation of mavacamten therapy (LVEF < 50%) was not observed in any of the study participants.

**Figure 1:**
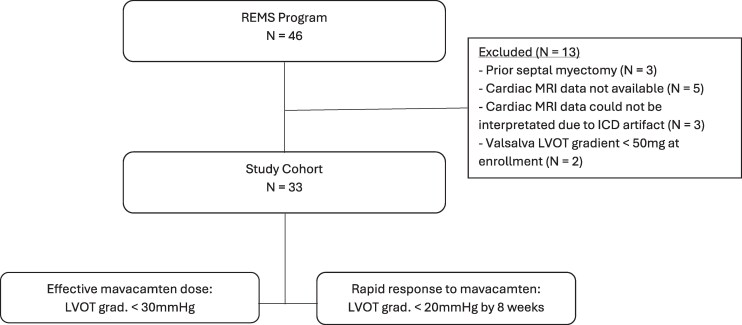
CONSORT diagram derivation of study cohort for end-point analyses: (i) effective mavacamten dose to achieve LVOT gradient <30 mmHg determined after at least 12-weeks of therapy and (ii) rapid response status defined as achieving LVOT gradient o zf <20 mm Hg by 8-weeks of therapy. Thresholds gradients for effective mavacamten dose and rapid response were chosen based on thresholds defined in the REMS protocol.

A total of 13 patients were excluded from the study for the following reasons: prior septal myectomy (*N* = 3), no pre-enrolment cardiac magnetic resonance imaging study (CMR) (*N* = 5), or with a CMR that could not be interpreted because of implantable cardioverter defibrillator artefact (*N* = 3). Patients with prior septal myectomy were excluded as these procedures are commonly performed with concurrent modification of the AMVL. Additionally, patients with a resting and Valsalva LVOT gradient of <50 mmHg at the time of enrolment were excluded (*N* = 2) (*Figure 1*). One of these patients was started on mavacamten at 5 mg daily for a provokable outflow tract gradient >50 mmHg after exercise. The second patient was started on mavacamten at 2.5 mg daily for exertional symptoms and a provokable gradient >30 mmHg after exercise.

### Imaging assessments

Echocardiograms were performed in accordance with contemporary American Society of Echocardiography (ASE) guidelines.^18^ LVEF, left-ventricular-global longitudinal strain (LV-GLS), LVOT gradients at rest and with Valsalva, right-ventricular (RV) systolic function and tissue-doppler S’ were assessed in accordance with ASE guidelines.^19,20^ MR severity was reported qualitatively in accordance with ASE guidelines as quantitation may be challenging given jet eccentricity.^21,22^ Severity of SAM of the AMVL and/or subvalvular apparatus was assessed from the parasternal long-axis (PLAX) view using M-mode and visual assessment. Severe SAM was defined by septal contact of the AMVL or chordal apparatus during systolic phase; moderate in cases in which the AMVL was measured within 1 cm of the septum; and mild if SAM of the AMVL was present but measured >1 cm from the septum. AMVL length was measured in the PLAX view during diastolic phase. AMVL measurements were made independently by two investigators, and the same two investigators re-measured AMVL at 6-week intervals in a blinded fashion for intra- and interobserver variability. For intraobserver variability, the mean absolute differences were 1.2 mm ± 0.82 mm and 0.93 mm ± 0.66 mm for each of the investigators, respectively. For interobserver variability, the mean absolute difference was 1.1 mm ± 0.84 mm.

Cardiac MRI (CMR) studies were performed according to a standard HCM protocol to optimize images for assessment of the LVOT, mitral valve and subvalvular apparatus. Studies were performed on 1.5T or 3.0T scanners (Siemens, Philips, GE, and Toshiba). Measurements of the LVOT diameter, anterior and posterior mitral valve leaflet length, bifid or multiheaded papillary muscles, and anteroapically displaced papillary muscles were performed by investigators blinded to clinical data. Anteroapically displacement of the papillary muscle was determined by oblique orientation of the papillary muscle bulk with insertion at or near the apex. AMVL length was measured in the 3-chamber view during diastolic phase. AMVL measurements were made independently by two investigators, and the same two investigators re-measured AMVL at 3-month intervals in a blinded fashion for intra- and interobserver variability. For intraobserver variability, the mean absolute differences were 1.1 mm ± 0.74 mm and 0.83 mm ± 0.59 mm for each of the investigators, respectively. For interobserver variability, the mean absolute difference was 0.6 mm ± 0.42 mm.

### Statistical analyses

Continuous data were summarized as median (quartile 1, quartile 3), and categorical data were expressed as *n* (%). Between group comparisons were performed with Fischer’s exact tests for categorical variables and Wilcoxon or Kruskal-Wallis rank-sum tests for continuous variables, as appropriate. Multivariable logistic regression was performed with a mavacamten dose of 5 mg daily as the dependent variable. Given the small sample size, adjustment was performed for AMVL length, age, and sex. A two-sided *P* < 0.05 was considered to indicate statistical significance. All analyses were performed with R Core Team (2023) (R Foundation for Statistical Computing, Vienna, Austria. https://www.R-project.org/). Data and analyses are available upon reasonable request to the authors. The Institutional Review Board at Northwestern University approved this study with a waiver of the need for informed consent.

## Results

### Effective mavacamten dose

Among patients who achieved resolution of LVOT gradients on low (≤5 mg, *n* = 13), intermediate (10 mg, *n* = 12), and high (15 mg, *n* = 8) doses of mavacamten, patients on low dose tended to be older compared with patients on intermediate and high doses (*Table 1*). At baseline, nearly all patients were treated with a beta-blocker or a non-dihydropyridine calcium-channel blocker (*n* = 31), and all patients had either NYHA Class II or III functional status.

**Table 1:** Baseline clinical characteristics stratified by final mavacamten dose

	5 mg/day(*n* = 13)	10 mg/day(*n* = 12)	15 mg/day (*n* = 8)	*P*-value
Age (years)	66.17 [58.01, 75.55]	57.98 [54.15, 64.70]	53.14 [45.79, 61.95]	0.046
Female sex	9 (69.2)	5 (41.7)	4 (50.0)	0.368
BMI	28.70 [23.50, 33.30]	28.30 [26.80, 37.33]	38.83 [32.48, 41.23]	0.115
White race (%)	9 (71.4)	11 (91.7)	6 (75.0)	0.2
Systolic blood pressure	132.00 [116.00, 137.00]	125.50 [120.00, 148.50]	129.50 [121.50, 140.50]	0.994
Atrial Fibrillation	2 (15.4)	4 (33.3)	0 (0.0)	0.157
Hypertension	8 (61.5)	7 (58.3)	6 (75.0)	0.735
Diabetes mellitus	3 (23.1)	3 (25.0)	0 (0.0)	0.307
Implantable cardioverter-defibrillator	1 (7.7)	4 (33.3)	1 (12.5)	0.225
Beta-blockers	11 (84.6)	11 (91.7)	6 (75.0)	0.595
Diltiazem or Verapamil	2 (15.4)	1 (8.3)	3 (37.5)	0.24
Disopyramide	2 (15.4)	2 (16.7)	1 (12.5)	0.968
Prior Alcohol Septal Ablation	2 (15.4)	0 (0.0)	0 (0.0)	0.194
NYHA Class III	5 (38.5)	3 (25.0)	0 (0.0)	0.136

Expressed as *n* (%) or median [quartile 1, quartile 3].

Baseline echocardiographic evaluation demonstrated patients had preserved-to-hyperdynamic systolic function, increased interventricular septal thickness, and elevated Valsalva-induced gradients across the LVOT tract among dosing groups, all in keeping with obstructive HCM (*Table 2*). A majority of patients had severe SAM (72%), and approximately two-thirds of patients had mild or greater mitral regurgitation (*n* = 26). LVOT diameter was smaller in patients on low-dose mavacamten, though numerically larger for patients on intermediate compared with high-dose mavacamten. LV-GLS was similar between dosing groups. RV systolic function was normal for all patients in the study.

**Table 2:** Baseline imaging characteristics stratified by final mavacamten dose

	5 mg/day(*n* = 13)	10 mg/day(*n* = 12)	15 mg/day(*n* = 8)	*P*-value
Left ventricular ejection fraction (%)	71.00 [68.00, 76.00]	69.50 [65.75, 72.00]	72.00 [71.75, 75.00]	0.402
Valsalva-induced left ventricular outflow tract gradient (mmHg)	81.00 [62.00, 84.60]	104.45 [99.50, 126.53]	59.90 [55.50, 90.12]	0.037
LV GLS (%)	−18.4 [−21.7, −17.3]	−18.2 [−19.68, −14.25]	−17.95 [−18.48, −15.80]	0.44
Systolic anterior motion				0.60
None	0 (0.0)	1 (8.3)	0 (0.0)	
Mild	2 (15.4)	0 (0.0)	0 (0.0)	
Moderate	2 (15.4)	1 (8.3)	1 (12.5)	
Severe	9 (69.2)	9 (74.7)	6 (75)	
Absent data	0 (0.0)	1 (8.3)	1 (12.5)	
Mitral regurgitation				0.62
Trace	3 (23.1)	3 (25.0)	1 (12.5)	
Mild	7 (53.9)	5 (41.5)	6 (75.0)	
Moderate	2 (15.4)	4 (66.8)	1 (12.5)	
Severe	1 (7.7)	0 (0.0)	0 (0.0)	
Interventricular septal thickness	17.5 [16.1, 18.5]	20.0 [16.5, 20.4]	19.0 [16.0, 22.0]	0.68
Left-ventricular outflow tract diameter	21.70 [20.30, 24.00]	24.50 [22.75, 25.80]	23.00 [21.00, 23.00]	0.048
Anterior mitral valve leaflet length (MRI)	20.00 [18.50, 20.80]	23.30 [22.45, 26.10]	25.45 [24.20, 26.85]	<0.001
Anterior mitral valve leaflet length (Echo)	*n* = 12 19.00 [18.00, 20.50]	*n* = 11 24.00 [21.50, 26.50]	*n* = 7 26.00 [24.50, 27.00]	0.003
Posterior mitral valve leaflet length	14.40 [10.70, 15.40]	16.40 [13.55, 18.75]	16.00 [15.52, 16.60]	0.083
Anterolateral papillary muscle area	148.80 [136.00, 169.00]	190.25 [174.45, 244.98]	211.05 [165.42, 239.80]	0.16
Bifid or multiheaded papillary muscles	9 (69.2)	7 (58.3)	6 (75.0)	0.718
Anteroapically displaced papillary muscles	7 (53.8)	10 (83.3)	7 (87.5)	0.142
RV systolic function (qualitative)				0.34
Normal	12 (92.3)	12 (100.0)	7 (87.5)	
Not seen	1 (7.7)	0 (0.0)	1 (12.5)	
RV S’	13.10 [12.68, 14.25]	13.35 [12.30, 14.77]	15.00 [11.45, 15.50]	0.821

Expressed as *n* (%) and median (quartile 1, quartile 3). Echocardiographic data was used to assess baseline left ventricular ejection fraction, Valsalva-induced LVOT gradient, SAM, mitral regurgitation severity, AMVL length, and right ventricular systolic function parameters. Cardiac MRI data was used to define all other measurements.

**Figure 2:**
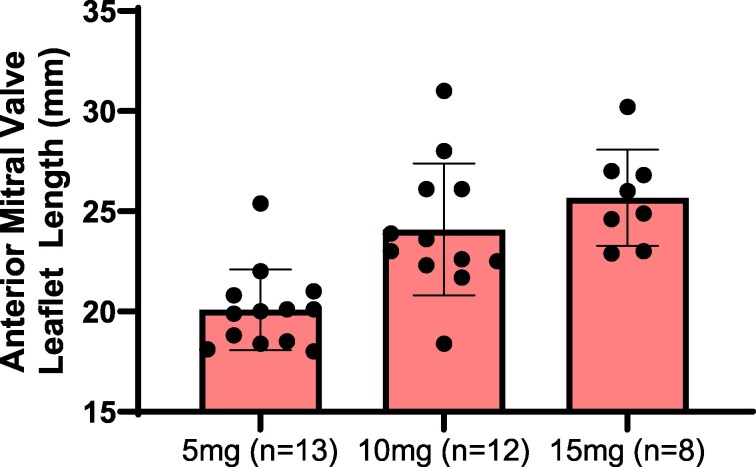
Box-whisker plot of AMVL length (mm) measured by cardiac MRI and stratified by final mavacamten dose. AMVL length was shorter in patients on a lower final dose of mavacamten [5 mg: 20.0 (18.5, 20.8) mm, *n* = 13; 10 mg: 23.3 (22.5, 26.1) mm, *n* = 12; 15 mg: 25.5 (24.2, 26.9) mm, *n* = 8] (*P* < 0.001).

AMVL length measured by CMR was shorter in patients on low-dose [20.00 (18.50, 20.80) mm], compared with patients on an intermediate [23.30 (22.45, 26.10) mm] and high-dose [25.45 (24.20, 26.85) mm] of mavacamten (*P* < 0.001) (*Figure 2*). After adjusting for age and sex, a mavacamten dose of 5 mg was associated with a shorter AMVL length (*β* = −0.08, *P* = 0.003). No other associations were noted between mitral valve and subvalvular apparatus abnormalities with the mavacamten dose required to achieve resolution of the elevated LVOT gradient.

AMVL length measurements assessed from available echocardiography data (low-dose, *n* = 12; intermediate, *n* = 11; high, *n* = 7) corroborated findings made by cardiac MRI (see Supplementary data online, *Figure S1*). AMVL was shorter in patients on low-dose [19.00 (18.00, 20.50) mm], compared with patients on intermediate [24.00 (21.50, 26.50) mm] and high-dose [26.00 (24.50, 27.00) mm] mavacamten (*P* < 0.003).

At most recent follow-up, LVEF and LVOT gradient were numerically lower and comparable across dosing groups. No patients in the study over the monitoring period experienced LV systolic dysfunction (LVEF < 50%) requiring discontinuation of therapy. The severity of SAM severity was improved and comparable across all dosing groups. Prior to initiating mavacamten, 72.7% of patients had severe SAM (low dose = 69.2%; intermediate = 74.7%; high = 75%), which improved to 15% (low dose = 23%; intermediate = 25%; high = 12.5%). LV GLS trended towards improvement with therapy, and RV systolic function remained normal. AMVL measurements were similar at follow-up imaging when compared with baseline data.

### Rapid response to mavacamten

**Figure 3:**
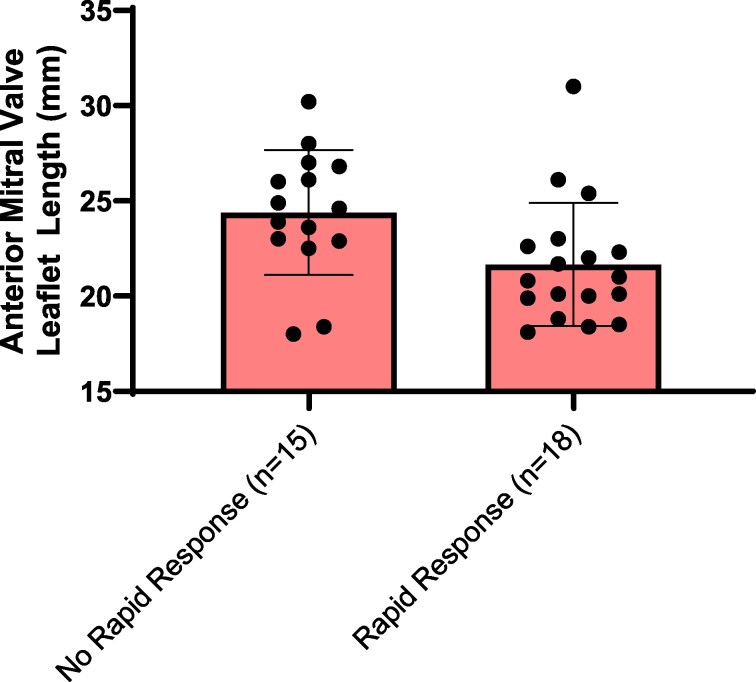
Box-whisker plot of AMVL length (mm) measured by cardiac MRI and stratified by rapid response to mavacamten. AMVL length was shorter in patients with a rapid response [20.9 (19.9, 22.5) mm; *n* = 18] compared with patients without a rapid response [24.9 (23.3, 26.5) mm; *n* = 15] (*P* = 0.006).

**Figure 4:**
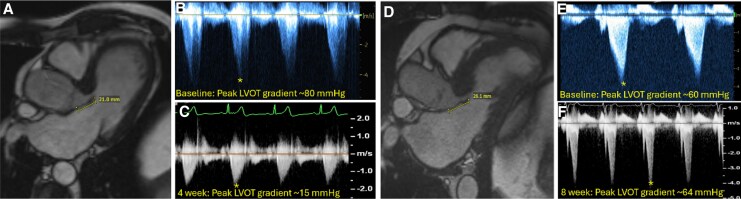
Case examples of a rapid responder (*A–C*) and a late responder (*D–F*) to mavacamten. (*A*) AMVL length (21 mm) measured by cMRI in mid diastole; (*B*) Peak LVOT gradient from baseline Echocardiogram; (*C*) Peak LVOT gradient after 4-weeks of Mavacamten 5 mg daily; (*D*) AMVL length (26.1 mm) measured by cMRI in mid diastole; (*E*) Peak LVOT gradient from baseline Echocardiogram; (*F*) Peak LVOT gradient after 8-weeks of Mavacamten 5 mg daily. *Peak velocities marked with asterisk.

Overall, 18 patients (54.5%) had a rapid response to mavacamten. In these patients, AMVL length was shorter among patients with rapid response compared with counterparts without a rapid response as determined by cardiac MRI [20.9 (19.9, 22.5) mm vs. 24.9 (23.3, 26.5) mm, *P* = 0006] (*Figure 3*). Similarly, AMVL was shorter among rapid responders as assessed by echocardiography [19.5 (18.0, 22.3) mm vs. 25.5 (21.8, 27.0) mm; *n* = 15] (see Supplementary data online, *Figure S2*). There was no association between starting gradient and rapid response. Representative cases of patients with and without a rapid response to mavacamten are shown in *Figure 4*.

## Discussion

Myosin-inhibitors have emerged as an effective therapy to improve LVOT gradient, symptom burden, and physical functioning in patients with obstructive HCM.^13,23^ However, underlying structural cardiac features associated with a differential response to myosin-inhibition have not been evaluated in clinical trials. With echocardiograms and dose-titration mandated at specific times after initiation of mavacamten, the REMS program provides an opportunity to evaluate associations with response to mavacamten in a standardized fashion. Specifically, observational studies can be performed in a real-world clinical practice with echocardiographic assessments and medication titration that mirrors clinical trials.

Two key observations were made in this study. First, among patients with obstructive HCM, a shorter AMVL length was associated with a rapid response to mavacamten therapy and a lower final dose of mavacamten to effectively reduce the elevated LVOT gradient. Second, the association of AMVL length with a lower dose of mavacamten remained significant after adjusting for age and sex.

Of note, interventricular septal thickness was not associated with a rapid response to mavacamten or the final mavacamten dose. This finding supports the concept that myosin-inhibitors relieve outflow tract obstruction by reducing force of ventricular contraction and systolic LVOT collapse irrespective of resting wall-thickness. Mechanistically, elongation of the AMVL can impede ejection across the LVOT independent of myocardial thickness and the force of ventricular contraction.^4–7^ Nonetheless, dose-escalation increased the likelihood of response regardless of AMVL length. This observation aligns with the complex interplay of obstructive structural features and haemodynamics in HCM. Flow drag is likely the dominant hydrodynamic force leading to LVOT obstruction,^14^ and reduction in this force with myosin inhibition will improve SAM of the mitral valve across a range of AMVL lengths.

A prior study of 121 patients with HCM without severe basal septal hypertrophy (≤ 1.8 cm) observed that each of AMVL length, abnormal chordal attachments, and bifid papillary muscle morphology were associated with LVOT obstruction.^5^ In our study, only AMVL length appeared relevant, and a few explanations are possible for this discrepancy. First, our study was small limiting power and degrees of freedom for discovery of other associations. Second, referral bias is possible; for example, patients with specific anatomic abnormalities that require intervention on the mitral valve or papillary muscles are likely to be referred directly to cardiac surgery. Finally, it is important to also consider the possibility that in patients treated with myosin inhibitors, AMVL length may be a predominant anatomic feature related to treatment response.

Key limitations of this study should be highlighted. This was a retrospective and single-centre study requiring all individuals have an interpretable CMR study for analysis. Additionally, as previously stated, the sample size was small thereby restricting the number of patient-factors that may be incorporated into a multivariable analysis. Of note, despite these limitations, the study findings are compelling as they draw on high-resolution and high-fidelity MR imaging and the observed association of AMVL with rapid response and effective dosing of mavacamten remains highly statistically significant. Nevertheless, these observations require validation in a larger and diversified study cohort.

In conclusion, this study is the first to evaluate specific features of the mitral valve and subvalvular apparatus that are associated with response to myosin inhibition in patients with obstructive HCM. Our findings suggest that AMVL length may be an important feature in understanding response to myosin inhibition. More broadly, this observation could represent an additional consideration in an individualized treatment approach to relieving LVOT obstruction among patients with HCM.

## Clinical perspectives

1. A shorter anterior mitral valve length is associated with a more rapid response and a lower effective dose of a myosin inhibitor in obstructive HCM.
2. Mechanistically, elongation of the AMVL can impede ejection across the LVOT independent of myocardial thickness and the force of ventricular contraction, although dose-escalation of myosin inhibition increases the likelihood of response regardless of AMVL length.
3. If this finding is confirmed in further studies, AMVL length might inform individualized decision making and prognosticate clinical response to myosin inhibition for patients with obstructive HCM.

## Supplementary Material

qyaf081_Supplementary_Data

## Data Availability

The primary data presented within this article has not been shared publicly to protect the privacy of study participants. Deidentified data and/or limited data can and will be shared at reasonable request to the corresponding author.

## Lead author biography

Danish Saleh is a physician-scientist and clinical fellow in Cardiology at Northwestern Memorial Hospital in Chicago IL. Danish has a special clinical interest in HCM and has worked under the mentorship of Dr. Lubna Choudhury at Northwestern. Danish attended Medical School at Tufts University in Boston, MA where he obtained his medical degree as well as a doctoral degree (PhD) in Neuroscience. Danish completed undergraduate training at the University of Pennsylvania in Philadelphia, PA where he graduated *Summa Cum Laude* in Biochemistry with *Phi Beta Kappa* Honours. Danish will be transitioning to independent practice in Cardiology as faculty at the University of Wisconsin–Madison.
