# Supplementary material for: Anterior mitral valve leaflet length and response to mavacamten in obstructive hypertrophic cardiomyopathy
Source: Eur Heart J Imaging Methods Pract. 2025 Jun 12;3(2):qyaf081. doi: 10.1093/ehjimp/qyaf081 (PMC12214459; doi:10.1093/ehjimp/qyaf081)
Supplement: qyaf081_Supplementary_Data [file qyaf081_supplementary_data.docx]

**Figure S1. Box-Whisker plot of anterior mitral valve leaflet length (mm) measured by echocardiography and stratified by final mavacamten dose.** Anterior mitral valve leaflet length was lower in patients on a lower final dose of mavacamten (5 mg: 19.0 [18.0, 20.5] mm, n = 11; 10 mg: 24 [21.5, 26.5] mm, n = 11; 15 mg: 26.0 [24.5, 27.0] mm, n = 7) (*p* = 0.003).

**Figure S2. Box-Whisker plot of anterior mitral valve leaflet length (mm) measured by echocardiography and stratified by rapid response to mavacamten.** Anterior mitral valve leaflet length was lower in patients with a rapid response (19.5 [18.0, 22.3] mm; n = 18) compared to patients without a rapid response (25.5 [21.8, 27.0] mm; n = 15) (*p* = 0.01).
